# Integrating multi‐omics data for target and biomarker discovery

**DOI:** 10.1002/alz.086331

**Published:** 2025-01-03

**Authors:** Matthias Arnold, the Alzheimer’s Disease Metabolomics Consortium

**Affiliations:** ^1^ Institute of Computational Biology, Helmholtz Zentrum München, German Research Center for Environmental Health, Neuherberg, Bavaria, Germany, Department of Psychiatry and Behavioral Sciences, Duke University, Durham, NC USA

## Abstract

**Background:**

Despite recent breakthroughs, Alzheimer’s disease (AD) remains untreatable. In addition, we are still lacking robust biomarkers for early diagnosis and promising novel targets for therapeutic intervention. To enable utilizing the entirety of molecular evidence in the discovery and prioritization of potential novel biomarkers and targets, we have developed the AD Atlas, a network‐based multi‐omics data integration platform. Through recent extensions, the AD Atlas provides a comprehensive database of high‐quality multi‐omics data that can be utilized for hypothesis‐free ranking of molecular markers and disease modules, as well as prioritization of potential novel targets and drug repositioning candidates.

**Method:**

We developed several graph‐based analysis tools from proximity searches to applications of artificial intelligence that can be applied to the AD Atlas. For prioritization of potential targets and biomarkers, we derived several network‐based metrics to score ‐omics entities for disease relevance by not only assessing evidence for a single marker but also for its functional neighborhood in the AD Atlas network. For disease module identification, we employed graph representation learning coupled with unsupervised clustering to extract functional modules as defined by the network structure. Finally, we propose an ensemble approach that enables weighted aggregation of drug repositioning predictions from both signature‐based and network‐based algorithms.

**Result:**

We demonstrate that the AD Atlas enables complex computational analyses for target and biomarker discovery and prioritization as well as *in silico* drug repositioning in AD. Using the integrated scores for prioritizing single targets and biomarkers for AD, we observe significantly higher relevance scores for genes that have been nominated as promising targets by the AMP‐AD consortium. We further find that extracted disease modules are enriched for specific AD‐relevant biological domains and can be ranked by disease relevance using similar graph‐based metrics. Finally, we demonstrate that drug repositioning candidates are significantly enriched for compounds that were or are being tested in clinical trials for AD.

**Conclusion:**

High‐quality, multi‐omics networks, such as the AD Atlas, enable exploitation of large‐scale heterogeneous data through computational applications for target, biomarker, disease module, and drug repositioning candidate discovery and prioritization.

